# Case, Illustrating Evil Effects of Veratrum Viride

**Published:** 1861

**Authors:** E. S. McIntire

**Affiliations:** Dallas City, Ills.


CASE—*Illustrating Evil effects of Veratum Viride.* By E. S. McIntire, *Dallas City*, *Ills.*

On the 31st of January last, Dr. T. C. Patterson and the writer, were requested by Dr. Newton of this place, to see his child Hattie, aged three years; we found her suffering from active inflammation of the parenchyma of the right lung; pulse 120, countenance flushed, breathing not much accelerated.

The doctor has been administering Syr. Scil. Com. sufficient to produce slight emesis, and as he said, improve the expectoration; we continued the syrup with the following:

℞ Hyd. Chlo. Mit. gr. X.
Div. Chart, No. V. S. One every two hours.

Feb. 1st. No improvement, bowels more freely during the night; pulse 130 full; cough dry and annoying; ordered the following:

℞ Tinc. Verat. Viride,

Syr. Scil. aa ℨ j.

Mix. S. Begin with two drops every three hours, increase one drop each dose.

Feb. 2nd. Pulse down to 70, vomiting had taken place before daylight this morning, countenance pale, pupils dilated, with the most alarming dyspnea, gave brandy with

℞ Pulv. Doveri,
Quinine Sulph. aa. gr. i.

to be given every two hours.

Afternoon. Dyspnea much relieved, arterial excitement appearing again, resumed again the Veratrum Viride as before.

Feb. 3d. The alarming respiration of the day before, again returned as soon as the characteristic effects of the Veratrum were manifest, and on its discontinuance was again left in good order. The case was further treated without the use of the Veratrum, and without any return of dyspnea.

Dr. A. Hard, of Aurora, in the "Report on the Medicinal Uses of Veratrum Viride" to the State Medical Society at its meeting 1860, says: "Veratrum promotes the secretions from the skin and mucous membranes, and from this cause emetic doses may be dangerous, particularly to young children The secretion from the bronchial mucous membrane being so great as to produce suffocation."

On this principle we account for the alarming symptoms in the case reported here, and caution all persons to observe the working of this potent, though excellent, remedial agent, especially in its administration to those of tender years.

Dallas City, *March* 12*th*, 1861.

